# Microstructural Characterization of the As-Cast and Homogenized Al-Cu-Mg-Ag Alloy

**DOI:** 10.3390/ma16010433

**Published:** 2023-01-03

**Authors:** Haitao Lin, Kai Zhu, Qilong Liu, Lifang Chen, Zhengan Wang, Xiwu Li

**Affiliations:** 1 Southwest Aluminum (Group) Co., Ltd., Chongqing 401326, China; 2 State Key Laboratory of Nonferrous Metals and Processes, GRINM Group Co., Ltd., Beijing 100088, China; 3 GRIMAT Engineering Institute Co., Ltd., Beijing 101407, China; 4 General Research Institute for Nonferrous Metals, Beijing 100088, China

**Keywords:** Al-Cu-Mg-Ag heat-resistant aluminum, as-cast and homogenized microstructure, dendritic segregation, large-scale industrialized ingot

## Abstract

In this study, the as-cast microstructure and the evolution of the homogenized microstructure of large-scale industrialized Al-Cu-Mg-Ag heat-resistant aluminum alloy ingots were investigated by means of optical microscopy (OM), scanning electron microscopy (SEM), energy dispersive analysis (EDS), and differential scanning calorimetry (DSC). The results indicate that the dendritic segregation is evident in the ingot along the radial direction, and the grain boundaries are decorated with lots of net-shaped continuous eutectic structures. With the homogenization time extension and the homogenization temperature increase, the eutectic phases (i.e., the primary Al_2_Cu phase, the Al_2_CuMg phase, and the AlCuMgAg quaternary phase) at the grain boundaries gradually dissolve back into the matrix. Meanwhile, most of the dendritic grain boundaries gradually become sparse and thinner. Finally, it is found that the optimal homogenization regime of the Al-Cu-Mg-Ag alloy is 420 °C/5 h+480 °C/8 h+515 °C/24 h.

## 1. Introduction

Due to its high specific stiffness, high specific strength, excellent corrosion resistance and creep properties, low density, easy processing, and desired fatigue resistance, aluminum alloys have gradually become one of the most widely used structural materials in aerospace, rail transit, automobiles, ships, and buildings [1,2,3,4,5,6,7], especially in civil aviation, as a critical structural material, high-strength, high-toughness, and damage-resistant aluminum alloys account for 20% to 35% of the weight of the fuselage structure [8]. In recent years, the high cruising speed of aircraft, resulting in severe friction between the surface of the fuselage and the air, has increased the skin’s temperature significantly [9]. The more commonly used traditional 2xxx series heat-resistant aluminum alloys, such as 2219, 2618, and 2D70, have shown good heat resistance, but the long-term, stable service temperature of these alloys is limited (≤150 °C) [10,11,12]. Therefore, in order to ensure the safety of the aircraft operation and meet the requirements of the ultra-high-speed flight, it is particularly urgent to develop new heat-resistant aluminum alloys that can serve stably at higher temperatures. In the 1960s, related research already reported that adding the Ag element to the Al-Cu-Mg alloy can affect the aging precipitation behavior of the alloy [13,14]. It makes the alloy precipitate not only the Al_2_Cu phase but also the Ω phase. The Ω phase is a nanoscale strengthened phase with an ideal size that can exist stably at 200 °C during aging. Subsequent studies have shown that Al-Cu-Mg alloys with Ag added have ideal room temperature properties, excellent high-temperature properties, and broad application prospects in future ultra-high-speed aircraft [10,15,16]. Therefore, regulating the aging precipitation behavior of the strengthening phases (e.g. θ′(Al_2_Cu) and Ω) in Al-Cu-Mg-Ag alloys appears to be crucial. Optimizing the ratio of the main alloying elements in traditional Al-Cu-Mg-Ag alloys is an effective method that directly affects the number densities and morphologies of the precipitates to improve the mechanical properties of the alloys [17]. Adding rare earth (RE) elements, such as Sm, Y, Sc, and Er, into the Al-Cu-Mg-Ag alloys is also proven to be another effective way to enhance the mechanical properties of the alloys [18,19,20,21]. Compounds with excellent thermal stability formed by the addition of the RE elements, as well as the inhibition of precipitates from coarsening, are the main reasons for enhancing the mechanical properties of the alloys [18]. The finer the age-strengthening phases are, the more uniformly dispersed they are, and the better the mechanical properties of the alloys are. 

It is well-known that to make the precipitates fine and uniformly dispersed in the alloy matrix, uniform distribution and a low degree of segregation of the elements in the alloy matrix before the aging heat treatment should be guaranteed. Therefore, the homogenization heat treatment is essential for the aging-strengthened aluminum alloy, such as the Al-Cu-Mg-Ag alloy. The Al-Cu-Mg-Ag alloy ingot fabricated by the semicontinuous casting process naturally has a specific inhomogeneity in the structure and chemical composition distribution. Therefore, a heat treatment process is essential to homogenize the alloy ingot to weaken the inhomogeneous distribution of the alloying elements. Although lots of previous research has been conducted on homogenizing Al-Cu-Mg-Ag alloys, the research objects are mostly laboratory casting alloys [9,22,23,24,25,26]. The industrialized large-scale Al-Cu-Mg-Ag alloy ingots are chosen as research objects in this research. Additionally, the emphasis is placed on the evolution of those second phases inside the alloy during the homogenization process. The axial and radial component segregation is also discussed.

## 2. Experimental Section

**Figure 1:**
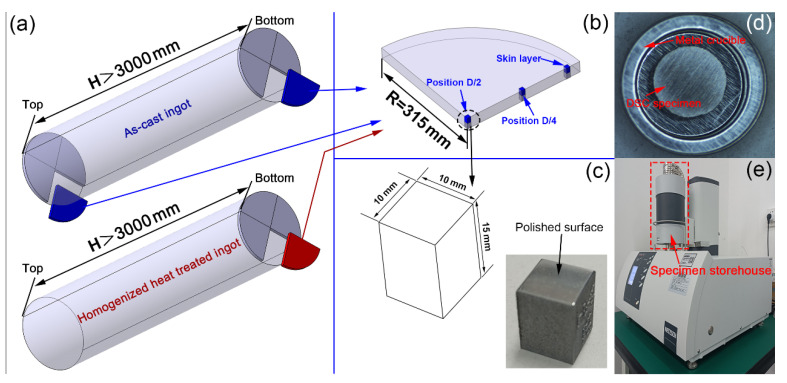
Schematic of the large-scale Al-Cu-Mg-Ag alloy ingots (**a**), sawed ingot slice (**b**), metallographic specimen (**c**), DSC sample (**d**), and the NETZSCH DSC 404 F3 equipment (**e**).

The Al-Cu-Mg-Ag alloy cylindrical ingot used in this research is produced by Southwest Aluminum Industry (Group) Co., Ltd. The schematic diagram of the shape of the ingot after head cutting, tail removal, turning, and peeling is shown in Figure 1a. In order to characterize the segregation of chemical components inside the ingot from two dimensions of height and radial direction, sawed slices with a thickness of 30 mm were processed from the top and bottom of the ingots. The schematic diagram of the slice structure is shown in Figure 1b. The samples used for microstructure characterization according to three specific positions (D/2, D/4, and the skin layer, as marked in Figure 1b) were sampled by the wire cut electrical discharge machining (WEDM) method along the radial direction of the slice. The three-dimensional size of the specimen is schematically shown in Figure 1c, as well as the physical photo. Here, it should be pointed out that the sampling plan of the specimen used for microstructure observation and characterization of the large-scale ingot after homogenization heat treatment is the same as shown in Figure 1a,b and will not be repeated.

The chemical compositions of the Al-Cu-Mg-Ag alloy investigated in this study were analyzed with ICP-AES by Agilent 725 (Agilent, Palo Alto, CA, USA) and then listed in Table 1. Zeiss Axiovert 200 MAT optical microscope (Zeiss, Oberkochen, Baden-Württemberg, Germany) was used to characterize the as-cast microstructure of the alloys at low magnification. A JEOL JSM 7001F scanning electron microscope (JEOL, Tokyo, Japan) working at a voltage of 20kV was used to observe the homogenized microstructure of the alloys. EDS (Oxford Instruments, Oxford, England) was used to assist the second phase analysis of the alloy. The initial melting temperature of the low-melting-point eutectic phase in the alloy was determined by NETZSCH DSC 404 F3 equipment (NETZSCH, Selb, Bavaria, Germany). The samples used for microstructure observation should be ground with 240#~5000# water-abrasive paper in sequence. Following that, the ground surfaces should be further polished with a particle size of 1 um diamond-abrasive paste until the scratches on the surfaces are not noticeable. Additionally, there is no need to etch the polished surface before observation. A metal disc with a diameter of Φ4 mm and a thickness of 1.4 mm (as schematically shown in Figure 1d) was cut by WEDM and ground by the 240#~1000# water-abrasive paper in sequence to remove all the oxide layers on the surface of the sample. Then, the polished sample was put into a metal crucible (as schematically shown in Figure 1d) and integrally moved into the sample chamber of the NETZSCH DSC 404 F3 equipment (as shown in Figure 1e). The sample was then heated from room temperature to 600 °C at a uniform heating rate of 10 °C/min in an inert atmosphere to capture the endothermic process.

## 3. Results and Discussion

### 3.1. As-Cast Microstructure

**Figure 2:**
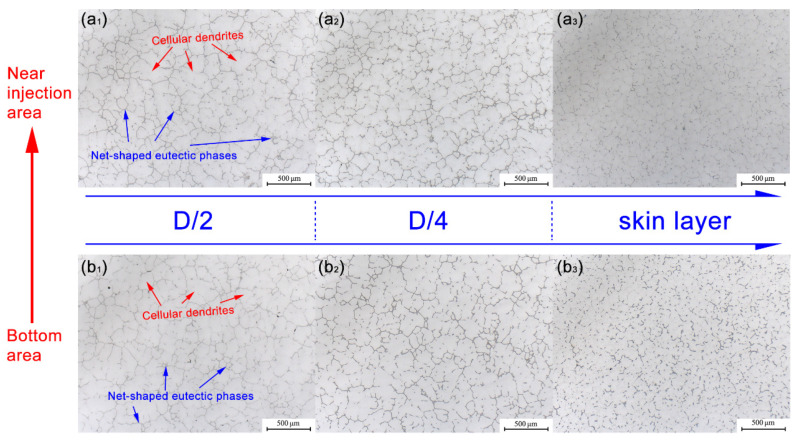
Metallographic microstructure of the as-cast Al-Cu-Mg-Ag alloy, respectively, corresponding to the D/2 (**a_1_**), D/4 (**a_2_**), and skin layer (**a_3_**) positions of the near injection area and the D/2 (**b_1_**), D/4 (**b_2_**), and skin layer (**b_3_**) positions of the bottom area.

Figure 2 is the metallograph representing the microstructure of those three specific positions of the top (near the injection end) and the bottom (close to the dummy end) areas of the large-scale ingot. Specifically, Figure 2a_1_–a_3_ were captured from the D/2, D/4, and skin layer positions of the ingot top area, respectively. Likewise, Figure 2b_1_–b_3_ represent the microstructures according to the D/2, D/4, and skin layer positions at the bottom of the ingot, respectively. Firstly, by longitudinal comparison of Figure 2a_1_/Figure 2b_1_, Figure 2a_2_/Figure 2b_2_, and Figure 2a_3_/Figure 2b_3_, it can be seen that the microstructure and morphology of the top and bottom typical positions (D/2, D/4, and skin layer) of the ingot are nearly same as each other and are all typical net-shaped as-cast structures. Taking the microscopic morphology of the top D/2 position of the ingot shown in Figure 2a_1_ as an example, it can be seen that the as-cast structure of the alloy mainly includes a developed, broad, and thick cellular dendritic structure (light color, marked by red arrows) and continuous, low-melting-point eutectic structure (grey color, marked by blue arrows) throughout grain boundaries. Secondly, it should be pointed out that compared with the ingot D/2 position and D/4 position, the morphology of the eutectic phase distribution at the grain boundaries within the skin layer area is changed into a certain intermittent distribution, which is shown in Figure 2a_3_,b_3_. The difference in the solidification behavior of liquid metal in different areas is the main reason for the above phenomenon because of the large diameter (> 630 mm). During the solidification process, the surface area of the ingot is directly connected to the inner wall of the mold cavity. A fast cooling rate accompanied by a high degree of subcooling makes the solute discharge during the rapid growth of dendrites, resulting in a depleted solute area in the surface/subsurface area of the ingot. In contrast, the ingot D/2 position and D/4 position developed into a solute-enrichment area. Finally, based on the above analysis, it can be deduced that there is a macrosegregation along the radial direction of the large ingot. 

Chemical composition analysis results of the main alloying elements about the typical locations (D/2, D/4, and skin layer) at the top and bottom areas of the ingot are shown in Table 1. It can be seen from the table that the content of the main alloying elements of the typical positions presents in a one-to-one manner. Moreover, it can be found that the distribution of the main alloy elements in the alloy ingot along the height direction of the ingot keeps consistent, and the degree of segregation is low. Additionally, by comparing the main element analysis results along the radial direction of the ingot, the content of the main alloying elements in the surface position appears significantly lower than that of the D/2 and D/4 positions. Therefore, a significant segregation phenomenon of the main alloy elements exists in the large-scale ingot along its radial distribution, being consistent with the microstructure analysis conclusions mentioned above. 

**Figure 3:**
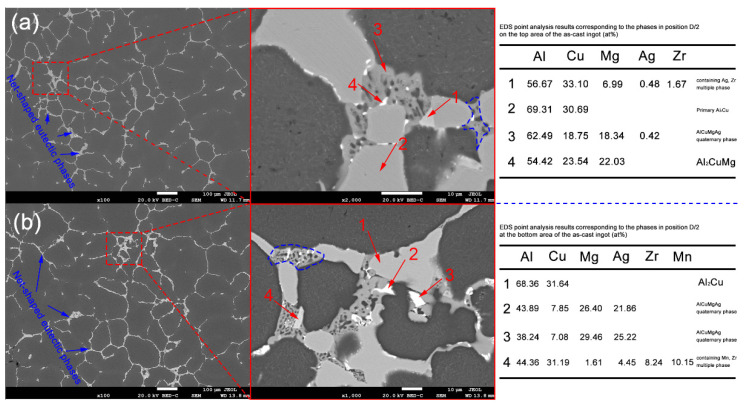
SEM-BSE images and EDS point analysis results of the Al-Cu-Mg-Ag alloy (**a**) D/2 position at the top of the ingot and (**b**) D/2 position at the bottom of the ingot.

To further clarify the composition of the grain boundary eutectic phase composition in the as-cast structure shown in Figure 2, the alloy’s microstructure, morphology, and phase composition at the D/2 position at the top and bottom of the ingot were characterized by means of SEM and EDS. Firstly, from the low magnification SEM-BSE photos shown in Figure 3, it can be seen that the as-cast microstructure of the alloy mainly includes cellular dendrites and eutectic phase at the grain boundary position, which is consistent with the OM results mentioned above. Secondly, the shape of eutectic phases at the grain boundary exhibits complex and diverse features, such as the honeycomb-like features, which are marked by the blue dashed box shown in Figure 3. Thirdly, in order to clarify the components of the low-melting-point eutectic phase at the grain boundaries, the points marked by the red arrows in Figure 3 are selected for the semiquantitative EDS analysis. The analysis results are listed in the attached table in Figure 3. It can be seen that these eutectic phases are mainly composed of the primary Al_2_Cu phase, the Al_2_CuMg phase, the AlCuMgAg quaternary phase, and the containing (Mn, Cr) phase. 

### 3.2. Homogenization Heat Treatment of the Large-Scale Al-Cu-Mg-Ag Alloy Ingots

Ingot homogenization treatment is a crucial heat treatment process in preparing age-strengthened aluminum alloys. After homogenization, the low-melting-point eutectic phases at the intragranular and grain boundaries can be effectively redissolved into the matrix, which improves the solid solubility of alloy elements in the matrix. However, the determination of the homogenization heat treatment closely depends on the eutectic phase at the grain boundary. In other words, the homogenization treatment temperature depends on the type and melting point of the eutectic phase, and the homogenization treatment time depends on the number of the eutectic phase and its dissolution rate. Generally, the higher the homogenization heat treatment temperature and the longer the treatment time is, the better the homogenization treatment of the ingot is. For the large-scale Al-Cu-Mg-Ag alloy ingot, the above analysis results have already shown a large number of low-melting-point eutectic phases at the grain boundaries in the as-cast structure, and the distribution of the elements in the matrix is not uniform. Therefore, the ingot must be subjected to a homogenization heat treatment to ensure that the alloying elements can be effectively dissolved back into the matrix. 

**Figure 4:**
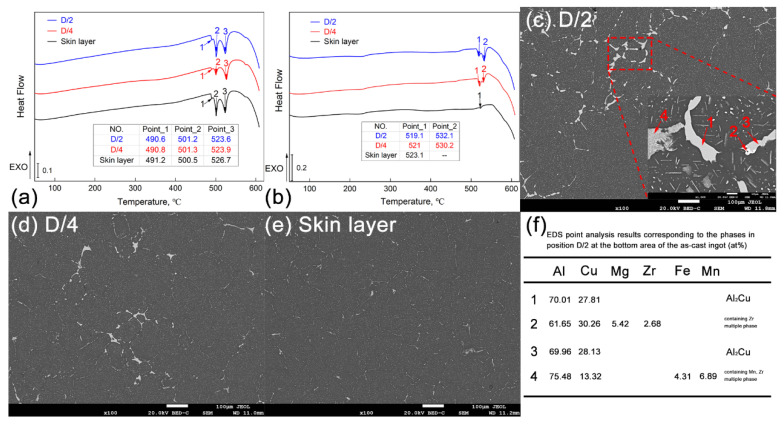
DSC analysis results of the as-cast Al-Cu-Mg-Ag alloy (**a**) and the homogenized Al-Cu-Mg-Ag alloy (**b**), SEM-BSE images and the EDS point analysis results of the homogenized Al-Cu-Mg-Ag alloy (**c**–**f**).

Differential scanning calorimetry (DSC) is the primary method of determining the homogeneous regime of alloys. As we all know, the melting process of the alloy is usually endothermic. When the Al-Cu-Mg-Ag alloy sample is tested by differential scanning calorimetry, there must be a potential difference at the melting point of the second phase, which then appears as a thermal effect peak in the DSC curve. Additionally, there is a corresponding relationship between the magnitude of the potential difference, the height of the thermal effect peak, and the amount of the second phase. The more the second phase is, the more significant the potential difference and the higher the thermal effect peak on the DSC curve is. In this study, samples extracted from the three specific positions (D/2, D/4, and skin layer) at the bottom of the ingot were selected for DSC analysis, and the results are shown in Figure 4a.

Firstly, it can be seen from Figure 4a that three noticeable endothermic reactions occurred near 490 °C, 500 °C, and 524 °C in the DSC curves corresponding to the above three typical positions. Based on the characteristic of the endothermic peaks, it is estimated that they are the melting temperature of the eutectic phase in the Al-Cu-Mg-Ag alloy. The first two endothermic peaks correspond to the low-melting-point eutectic phase (i.e., the Al_2_CuMg phase and the AlCuMgAg quaternary phase), and the third endothermic peak corresponds to the high-melting-point eutectic phase (the primary Al_2_Cu phase). For this, it can be determined that the homogenization heat treatment temperature of the alloy should not be higher than 524 °C. In addition, due to the addition of the Zr element in the alloy, a 420 °C/5 h low-temperature pretreatment for the ingot should be performed to facilitate the precipitation of Al_3_Zr particles, which have high thermal stability and play a crucial role in inhibiting the recrystallization and refining the grains. Therefore, taking the above DSC analysis results for the as-cast alloy and the extrapolation method into consideration, the P1 principle of the homogenization heat treatment for the large-scale ingot is developed and shown in Table 2.

Figure 4b shows the results of DSC analysis for the three typical locations (D/2, D/4, and skin layer) at the bottom of the alloy ingot after homogenization heat treatment. First of all, it can be seen from the figure that the two endothermic peaks corresponding to the low-melting-point eutectic phase in the DSC curve disappeared after the homogenization heat treatment of the alloy. It indicates that those low-melting-point eutectic phases (i.e. the Al_2_CuMg phase and the AlCuMgAg quaternary phase) have fully dissolved into the matrix after the double-stage homogenization heat treatment at 420 °C/5 h + 480 °C/8 h. However, the endothermic peak around 524 °C in the curve still exists, indicating that the high-melting-point eutectic phase (the primary Al_2_Cu phase) remains. Secondly, compared with the skin layer position, the endothermic peaks around 524 °C corresponding to the DSC curves of the D/2 and D/4 positions are higher and show a bimodal phenomenon. The difference in the as-cast microstructure of the three positions is the main reason for the difference in thermal effect peaks on the DSC curves. From the above analysis, it has already been proven that there is a macrosegregation along the radial direction of the large-scale Al-Cu-Mg-Ag alloy ingot.

Furthermore, the number of grain-boundary eutectic phases at the D/2 and D/4 positions is significantly more than that of the skin layer position. Therefore, when the ingot is integrally heat-treated through the same homogenization heat treatment principle, a lower thermal effect peak reasonably presents on the DSC curve due to the low number of the eutectic phases in the skin layer area. In contrast, the thermal effect peaks on the DSC curves corresponding to the D/2 and D/4 positions are higher. Air cooling condition for the large-scale ingot after the homogenization heat treatment accounts for the appearance of the bimodal phenomenon. A slower cooling rate resulting from the air cooling condition leads to some high-melting-point second phases with small sizes precipitating from the matrix to weaken the solid solubility and result in a pseudobroadening of the endothermic peak near 524 °C on the DSC curve. Figure 4c–e are low-magnification SEM images of typical positions (D/2, D/4, and skin layer) at the bottom of the ingot after homogenization heat treatment. It can be seen that the number of eutectic phases at the grain boundaries is significantly reduced after the treatment; however, the number of the residual second phases at the grain boundaries of the D/2 and D/4 positions is intuitively more than that at the skin layer position. The SEM characterization results match the DSC curve analysis results well. Further EDS point analysis is still conducted here for the sites marked by red arrows in Figure 4c to clarify the residual undissolved phases. Through the P1 homogenization heat treatment, the remaining second phase in the large-scale Al-Cu-Mg-Ag alloy ingot is mainly the Al_2_Cu phase, containing the Zr phase and containing the (Fe, Mn) phase, as shown in the results listed in Figure 4f. Additionally, the excess of the residual Al_2_Cu phase indicates that optimization for the homogenization heat treatment for this Al-Cu-Mg-Ag alloy is still needed. 

### 3.3. Optimization for the Al-Cu-Mg-Ag Alloy Homogenization Heat Treatment

**Figure 5:**
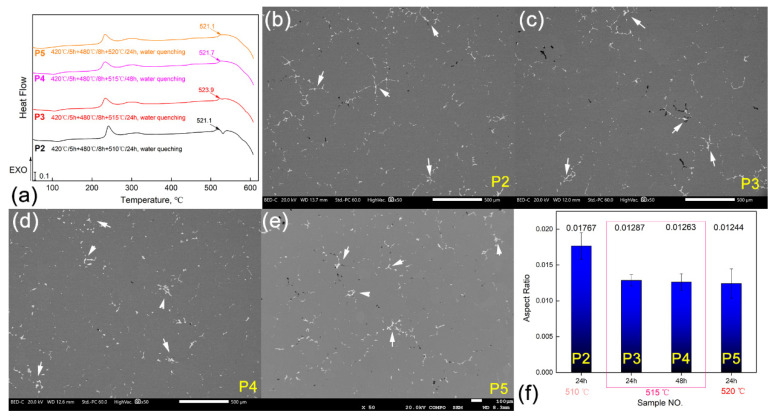
DSC analysis results of the Al-Cu-Mg-Ag alloy homogenized by P2, P3, P4, and P5 (**a**), SEM-BSE images and the EDS point analysis results of the homogenized Al-Cu-Mg-Ag alloy corresponding to the homogenization principles of P2, P3, P4, and P5 (**b**), (**c**), (**d**), (**e**), and (**f**), respectively.

The alloy of the D/4 position extracted from the bottom area of the ingot was chosen as the research object in this section for optimizing the homogenization heat treatment principle. The P2, P3, P4, and P5 principles shown in Table 2 are the new heat treatment principles that have been proposed. Here, it should be pointed out that, except for the cooling condition, the P2 and P1 principles shown in Table 2 are consistent. Additionally, the purpose of adding the P2 principle is to set up a basic comparison principle in the optimization process. The specific optimization process is carried out in the laboratory, and the temperature control accuracy of the heat treatment furnace is ±2 °C. Firstly, it can be seen from the DSC curves shown in Figure 5a that with the increase in the homogenization heat treatment temperature (510 °C→515 °C→520 °C), the value of the thermal effect peaks on the DSC curves gradually decreases, which means that the number of the remaining second phases in the alloy gradually decreases as the homogenization heat treatment temperature increases. However, the difference between the peaks of the thermal effect peaks on the DSC curves corresponding to the P3 and the P5 principles is not significant. Secondly, further prolonging the homogenization heat treatment time based on the P3 system has no significant effect on the redissolving impact of the second phase in the alloy, which also can be seen from the DSC curves shown in Figure 5a.

Figure 5b–e are the representative low-magnification SEM images of the Al-Cu-Mg-Ag alloy heat-treated by different principles. In the figures, it can be seen that a certain number of residual second phases (marked by the white arrows in the figures) still remained in the alloys after the solution heat treatments. An abundant copper element might be the main reason for this phenomenon, and our future work will place emphasis on optimizing the content of Cu element. To further quantify the effect of different homogenization principles on the redissolving impact of the second phase in the alloy, the area fraction of the residual second phase in the samples treated with the P2, P3, P4, and P5 regimes was also counted from more captured photos using the digital image method. The results are clearly shown in Figure 5f, which shows that the number of residual second phases in the alloy treated by the P2 principles is larger than those treated by the other three principles. Therefore, taking the control accuracy of the heat treatment furnace in the industrial production process into account to avoid overburning the alloy due to excessive temperature, this study suggests that the ideal homogenization principle for the Al-Cu-Mg-Ag alloy ingot is the P3.

## 4. Conclusions

(1) There are many dendrites and grain boundary eutectic phases, such as the primary Al_2_Cu phase, the Al_2_CuMg phase, the AlCuMgAg quaternary phase, and the containing (Mn, Cr) phase, in the as-cast structure of large-scale industrial Al-Cu-Mg-Ag aluminum alloy ingots. Compared with that along the height direction of the ingot, there is significant segregation along the radial distribution. The content of the main alloying elements in the skin layer position is lower than that in the D/2 and D/4 positions.

(2) After homogenization by the P1 principle, there is still a considerable amount of Al_2_Cu phase in the alloy. When the homogenization heat treatment temperature increases from 510 °C to 515 °C, the redissolving effect of the second phase is significantly improved. However, further turning the temperature up to 520 °C does not considerably affect the redissolving effect of the second phase.

(3) When the heat treatment temperature is 515 °C, continuing to prolong the homogenization heat treatment time also has little effect on the redissolution effect of the second phase in the alloy. Therefore, the P3 principle is an ideal homogenization heat treatment principle for the Al-Cu-Mg-Ag alloy in our research.

## Figures and Tables

**Table 1:** Results of the chemical composition analysis of the different typical positions in the ingot.

Positions	NO.	Element Type (w/%)					
		Mg	Mn	Cu	Ag	Zr	Al
Top area	D/2	0.96	0.62	5.30	0.62	0.11	Bal
	D/4	0.96	0.66	5.24	0.62	0.12	Bal
	Skin layer	0.80	0.60	4.14	0.53	0.13	Bal
Bottom area	D/2	0.92	0.61	5.20	0.60	0.11	Bal
	D/4	0.94	0.66	5.23	0.62	0.12	Bal
	Skin layer	0.82	0.60	4.43	0.54	0.12	Bal

**Table 2:** Various homogenization heat treatment principles.

NO.	Heat Treatment Parameters	Position
P1	420 °C/5 h + 480 °C/8 h + 510 °C/24 h, air cooling	D/2, D/4, Skin layer
P2	420 °C/5 h + 480 °C/8 h + 510 °C/24 h, water quenching	D/4
P3	420 °C/5 h + 480 °C/8 h + 515 °C/24 h, water quenching	
P4	420 °C/5 h + 480 °C/8 h + 515 °C/48 h, water quenching	
P5	420 °C/5 h + 480 °C/8 h + 520 °C/24 h, water quenching	

## Data Availability

Not applicable.
